# Airway Wall Remodeling in Childhood Asthma—A Personalized Perspective from Cell Type-Specific Biology

**DOI:** 10.3390/jpm11111229

**Published:** 2021-11-19

**Authors:** Lei Fang, Michael Roth

**Affiliations:** Pulmonary Cell Research/Pneumology, Department of Biomedicine/Internal Medicine, University & University Hospital Basel, Hebelstrasse 20, 4031 Basel, Switzerland; lei.fang@unibas.ch

**Keywords:** airway wall remodeling, asthma, epigenetic, bronchial epithelium, cell-cell interaction, inflammation, extracellular matrix, bio-markers

## Abstract

Airway wall remodeling is a pathology occurring in chronic inflammatory lung diseases including asthma, chronic obstructive pulmonary disease, and fibrosis. In 2017, the American Thoracic Society released a research statement highlighting the gaps in knowledge and understanding of airway wall remodeling. The four major challenges addressed in this statement were: (i) the lack of consensus to define “airway wall remodeling” in different diseases, (ii) methodologic limitations and inappropriate models, (iii) the lack of anti-remodeling therapies, and (iv) the difficulty to define endpoints and outcomes in relevant studies. This review focuses on the importance of cell-cell interaction, especially the bronchial epithelium, in asthma-associated airway wall remodeling. The pathology of “airway wall remodeling” summarizes all structural changes of the airway wall without differentiating between different pheno- or endo-types of asthma. Indicators of airway wall remodeling have been reported in childhood asthma in the absence of any sign of inflammation; thus, the initiation event remains unknown. Recent studies have implied that the interaction between the epithelium with immune cells and sub-epithelial mesenchymal cells is modified in asthma by a yet unknown epigenetic mechanism during early childhood.

## 1. Introduction

Airway wall remodeling is a persistent pathology in asthma, which is resistance to treatment. Tissue remodeling in the airways is the result of epithelial cell derangement, goblet cell hyperplasia, increased airway smooth muscle cells, thickening of the basal membrane, increased neovascularization in the sub-epithelial cell layers, and increased deposition of various extracellular matrix components [1]. It is currently unknown if specific structural changes characterize asthma pheno- and endo-types [2,3]. Moreover, none of the drugs used for asthma therapy show any reducing effect on airway wall remodeling [4]. In adult asthma patients, bronchial thermoplasty is the only therapy reducing airway wall remodeling in some patients with severe asthma, but not all [5]. The reason why some patients respond to heat therapy and others do not remains unknown; furthermore, this therapy cannot be applied to children. The cause of airway wall remodeling is not well understood, and most studies have investigated this pathology in adult asthma. Furthermore, the structure of airway wall remodeling might be divided into pheno- and endo-types, which may characterize specific asthma types. 

In 2017, the American Thoracic Society stated that the role of airway wall remodeling in asthma is insufficiently understood and needs to be further investigated [6]. Neither its origin nor its contribution to asthma is known, and the hypothesis that chronic inflammation is the only cause of airway wall remodeling has been challenged in recent years [7,8,9,10,11]. In an official workshop report of the American Thoracic Society, it was concluded that air pollution is most likely to pre-condition a child’s lungs to develop asthma or COPD later in life. Long-term exposure to fine particulate matter (PM) and ozone initiated airway wall remodeling, which can either lead to asthma or COPD [12].

**Figure 1:**
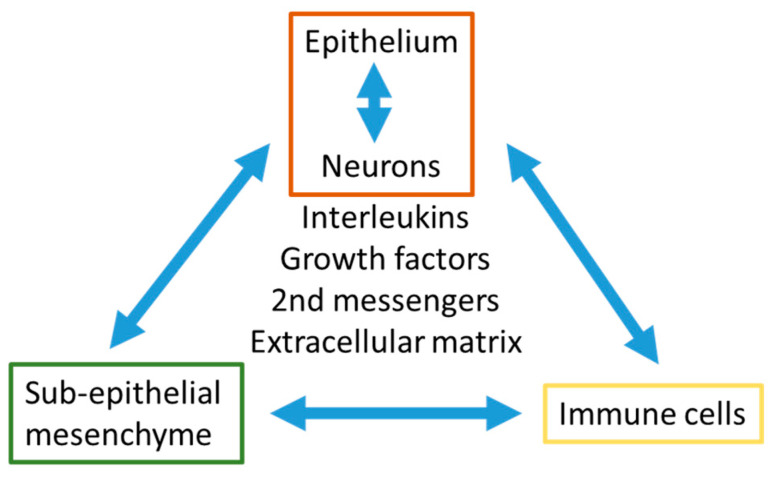
The interactions between the epithelium and neurons with sub-epithelial mesenchymal cells (airway smooth muscle cells, fibroblasts, myo-fibroblasts) and immune cells have to be seen as a unit, which regulates its function by secreted interleukins, growth factors, secondary messenger peptides, and the local composition of the extracellular matrix.

It has been long hypothesized that airway wall remodeling in childhood asthma is progressing over the duration of the disease and is caused by chronic inflammation. Referring to some studies on the causes of airway wall remodeling during embryogenesis and early childhood [10,11,12], this hypothesis has been challenged [13,14,15,16,17]. The earlier assumption that asthma, atopy, and bronchial hyper-reactivity are caused by genetic modifications of immune regulatory proteins was not confirmed [13,18]. Nearly twenty years ago, the significantly increased thickness of the lamina reticularis due to increased collagen deposition correlated with the expression of the epidermal growth factor receptor, but without eosinophilic infiltration in children with asthma [14]. In a primate model, it has been reported that post-natal exposure to ozone or house dust mite allergens resulted in a lasting modification of the airway structure including epithelial hyperplasia, increased goblet cell number, increased size of smooth muscle bundles, reduced differentiation of the basement membrane, and hyper-reactivity. Furthermore, the distribution of nerve cells within the epithelium was modified and the airway vascularization, as well as the immune response, were altered [15]. The thickening of the lamina reticularis in asthma was later linked to the disturbed communication between epithelium and sub-epithelial mesenchymal cells [16]. As reviewed by Veres et al. [19], airway inflammation and remodeling involved the communication between immune cells and tissue forming cell types including the epithelium, neurons, and sub-epithelial cells through neuropeptides (Figure 1). The release of neuropeptides may occur through epigenetic events triggered by environmental factors during embryogenesis and early childhood, which may trigger airway wall remodeling [10,12]. Importantly, such events can be inherited and, thus, mimic genetic traits [18]. 

This review summarizes the current knowledge on airway wall remodeling in asthma with a specific focus on childhood asthma. The objective of this review is to highlight the gaps of knowledge for understanding airway remodeling in childhood asthma, and thereby stimulate future studies in this important topic. Asthma cannot be cured unless the cause and the mechanisms of airway remodeling are understood. 

## 2. Early Events Leading to Asthma and Airway Wall Remodeling in Children

Over the past decades, despite the decline in asthma mortality, asthma morbidity is on the rise significantly without a clear reason. In a longitudinal study following 180 children from birth to the age of 36 years, it was indicated that asthma was initiated during embryogenesis and presents as a deficit of lung function [20]. Furthermore, the nerve density and airway reactivity in adult age was linked to IL-5 exposure during embryogenesis [21,22]. 

Today, it is widely assumed that poor indoor air quality and exposure to allergens are the main reasons for developing childhood asthma [23,24,25,26,27,28]. Rhinovirus infection at a young age presents another risk factor to develop asthma later in life [29]. Interestingly, rhinovirus infection significantly upregulated proteins that are known to regulate extracellular matrix (ECM) degradation, while proteins that stimulate *de novo* synthesis of the ECM were less affected. It should be noted that preterm born children were even more affected [29]. In atopic children, respiratory syncytial virus (RSV) infection resulted in altered interferon synthesis by nasal epithelial cells, but not by tracheal epithelial cells [30]. Rhinovirus infection upregulated the expression of the epithelial cell protein, CDHR3 (cadherin-related family member 3), and thereby altered the integrity and function of ciliated epithelial cells [31]. CDHR3 was associated with remodeling of epithelial cells by a large-scale genome-wide study comparing 1173 severe asthmatic to 2522 non-asthmatic children [32]. 

**Figure 2:**
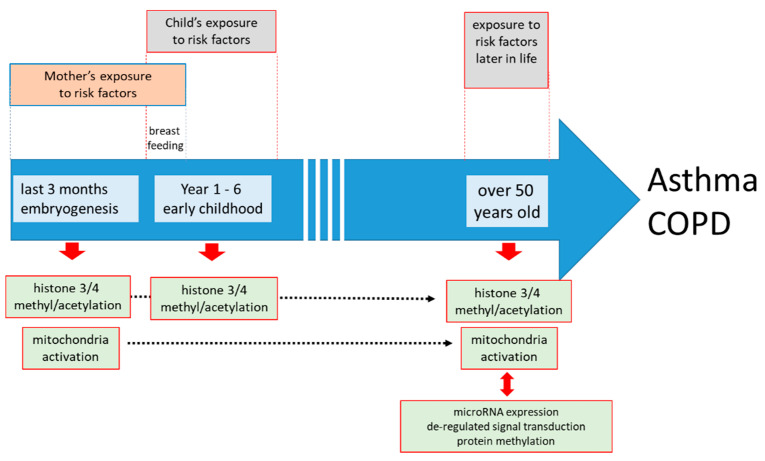
The sequence of known epigenetic events during embryogenesis and childhood predisposing the lung to develop chronic inflammatory lung diseases later in life. Today, it is unknown if the modification of specific genes leads to specific diseases, or if it is the nature of the second exposure to risk factors that is decisive for the development of asthma or COPD.

Other studies suggested that the many pathologies that characterized asthma might be explained by epigenetic imprinting of the developing lung during embryogenesis and early childhood [17,18]. The best studied epigenetic mechanisms related to asthma are histone modification, DNA methylation, and altered expression of specific micro RNAs (Figure 2). These epigenetic events occur in a cell type-specific manner and may not be presented in all cell types forming the airways. Moreover, the mechanisms of how such epigenetic events become permanent and even inheritable are not clear. 

Exposure to allergens and air pollution early in life correlated with an increased susceptibility to develop asthma and chronic obstructive pulmonary disease (COPD) later in life [33]. Volatile organic compounds such as propylene glycol contained in wall paint, cosmetics, and e-cigarettes have been reported to cause airway wall remodeling in children [27]. Comparing the airway wall structure between 39 children with allergic asthma to 21 children with allergic rhinitis and 20 healthy controls indicated a disease-specific type of tissue remodeling in the upper airways in asthmatic children [34]. In a retrospective study collecting data over 10 years, inner city children with asthma were grouped according to lung function [28]. The data analysis suggested that forced expiratory volume in 1 s predicted (FEV1) and forced vital capacity (FVC) <80% at young age indicated long-term reduced lung function. Additional risk factors were African American ethnicity and male gender. The authors concluded that the current asthma therapies do not affect airway wall remodeling in children with asthma. In another study, structural changes of the airways were investigated in biopsies obtained from 53 children with wheezing and compared to 45 children without wheezing [26]. Prolonged exposure to PM10 (10 microns) resulted in increased thickness of the basement membrane and eosinophilic inflammation in wheezing children. Increased thickness of the basal membrane was linked to airway smooth muscle cell remodeling and mitochondria mass/activity in pre-school children, who later developed asthma [35]. In addition, this study linked the mitochondria pathology and proliferation to increased intracellular calcium levels in regard to airway wall remodeling. 

## 3. The Maturation of the Epithelium and the Basement Membrane in Childhood and Its Link to Asthma

In adults, Grainge et al. [36] demonstrated that inflammation independent bronchoconstriction is sufficient to alter the airway structure, suggesting that mechanical forces might initiate remodeling. This led to the idea that pathologies of singularities such as the epithelium or airway smooth muscle cells are insufficient to explain what happens in asthma to the airway tissue structure [9,37]. However, the epithelium seemed to play a major role as a regulator.

Increased basement membrane thickness is one of the most common pathologies of asthmatic airway wall remodeling. Biopsy-based studies demonstrated thickened basement membrane in the airways of school children with severe and moderate asthma, and preschool children with recurrent wheezing [38,39,40]. Furthermore, a three-year follow-up study indicated that during infancy, the thickness of the basement membrane correlated with reduced lung function at an older age [41]. In a systematic postmortem study (47 preterm babies, 40 children, and 23 adults), it was reported that the epithelium and the basement membrane develop in parallel, being visible after gestation week 30 [16]. The thickness of the basement membrane increased rapidly until the age of 3 (3.5μm), and then slowed down until 17 years of age. This then remained stable over several years before declining after the age of 30. Interestingly, the thickness of the epithelium and the basement membrane correlated at younger age, and this correlation was lost after the age of 30 [16]. Therefore, it is important for studies on airway wall remodeling to apply age-adjustment in the analysis.

In addition to this, the airway epithelium may control airway remodeling after mechanical stimulation, and it had been suggested that airway wall remodeling might be the result of frequent bronchial constrictions, which cause mechanical stress to the tissues [9]. This hypothesis suggested that remodeling does not have to occur in the same area where bronchial constriction took place. In a cohort of 49 children with wheezing and airway hyper-responsiveness, it was indicated that remodeling was independent from inflammation [10]. Furthermore, it was suggested in a mouse model that the disruption of the airway epithelium during embryogenesis is a key event that imprints the lungs to develop asthma and airway wall remodeling later in life [42]. 

In children with therapy resistant asthma, bronchoscopic investigation and brushing suggested that wound repair of the epithelium was delayed compared to pre-school children with wheezing [43]. Epithelial cells from these patients showed reduced ability to overcome respiratory syncytial virus infection despite their increased secretion of anti-viral cytokines. In bronchial tissues, these children showed increased deposition of collagen. In a mouse model, IL-6 deficiency resulted in dysregulation of the tight junction between epithelial cells, reduced protection against allergic inflammation, and increased production of TGF-β, the most powerful stimulator of tissue remodeling [44]. 

## 4. Sub-Epithelial Mesenchymal Cells and Their Role in Airway Wall Remodeling

The role of airway smooth muscle cells in asthma-associated airway wall remodeling has been demonstrated in adults [45,46]. The cause of this pathology remains unclear, but the increased proliferation of airway smooth muscle cells is maintained in isolated cells of asthma patients [47,48]. Despite many studies investigating the reason of this increased proliferative capacity of airway smooth muscle cells, there has been no concept of how the different factors are linked to one another. In adult asthma, histones and DNA methylation have been linked to remodeling [49,50]. These findings might be linked to the lack of certain transcription factors such as C/EBP-α [51,52]. C/EBPs and methylation are controlled by a large range of microRNAs [53], which also play a role in asthma [54]. In regard to childhood asthma, there is increasing evidence that epigenetic events during embryogenesis and early in life lastingly affect DNA and histone methylation, microRNA expression, and cellular activity [55,56,57]. Moreover, risk factors in the environment of the mother during embryogenesis have been shown to initiate epigenetic mechanisms, which become persistent during later life stages and set the lung to develop chronic inflammatory diseases [57,58]. 

During early childhood, breast feeding transfers beneficial factors, but also risk factors, from mother to child. Overall, it can be concluded that breast feeding has more beneficial effects and reduces the risk of childhood asthma [59,60]. However, it should be mentioned that under certain conditions, breast milk contains allergens and cytokines (IL-4, IL-5, IL-13, TGF-β, etc.) that might contribute to the development of asthma and airway wall remodeling [60]. The composition of breast milk is affected by environmental factors to which the mother is exposed and transferred to the child indirectly. Breast milk feeding may also have an effect on asthma by modifying the microbiome and the gastrointestinal mucosal immunity of the digestive system, which communicates with the respiratory system [61,62]. There is evidence that food during early childhood affects DNA methylation mainly of cytosine-guanine dinucleotides, and this epigenetic modification was higher in children fed with breast milk compared to children exclusively formula fed [63]. However, DNA methylation might not be the only epigenetic mechanism by which the precondition to asthma during childhood is regulated, and further studies are needed [64]. Some literature suggests that microRNAs can be transmitted from one cell type to another cell type via vesicles through the blood stream and, therefore, might present a new way of communication between the epithelium and sub-epithelial cells [65]. This transmission of information between different cell types might initiate airway wall remodeling.

Below the epithelium and the basement membrane follows a layer of mesenchymal cells, which consists of fibroblasts, myo-fibroblasts, and airway smooth muscle cells. Especially, the hypertrophy and hyperplasia of airway smooth muscle cells is considered to be a key pathology of asthma and a major contributor to airway wall remodeling [66]. In childhood asthma, smooth muscle cell remodeling has also been reported, but it was not clear if this is a link to inflammation or an independent pathology [67,68]. However, the discussion of if airway wall remodeling is also a characteristic of childhood asthma remains controversial and might be linked to asthma pheno-/endo-types [10].

Comparing the airway histology between 21 children with non-eosinophilic asthma to that of 34 children with eosinophilic asthma and 25 non-asthma controls, it was clearly demonstrated that airway remodeling was independent of eosinophilic inflammation, and asthma-relevant mediators such as IL-4, IL-5, and TGF-β [69]. Increased airway smooth muscle mass was reported in school children (median age 12) with severe and difficult to treat asthma [70], as well as in preschool children with recurrent wheezing (median age 7.8) [71]. Both studies indicated that airway remodeling in children is rather independent of Th2 inflammation. In 49 pre-school children with wheezing, the size of the airway smooth muscle inversely correlated with FEV1/FVC [72]. Interestingly, the increased smooth muscles in asthma might be a major source of pro-inflammatory asthma-relevant cytokines such as IL-13, IL-17, IL-22, IL-33, lymphopoietin, semaphorins, and CXC chemokines [71,72,73]. All these factors alone have been reported to either interfere with proliferation or are being released by airway smooth muscle cells [74,75,76,77,78,79,80]. 

Neo-vascularization had also been reported as a pathology of the sub-epithelial cell layers in children with asthma, but this might be rather a consequence of remodeling than a cause. Comparing the airway structure of 13 asthma patients (including children) to 12 controls, it was reported that asthma was significantly associated with increased thickness of vessel tunica intima, while the tunica media was not affected [81]. Furthermore, the increase of the vessel walls was independent of age, and was attributed to increased, deranged collagen deposition. In a cohort of 49 pre-school children (median age 10.9 years), 26 (72.2%) had persistent asthma and the vessel density negatively correlated with FEV1% predicted and FEV1/FVC [70]. Comparing the level of the angiogenesis stimulating factors including the vascular endothelial growth factor VEGF), a negative correlation with the duration of asthma was reported [82]. However, all asthmatics inhaled glucocorticoids, and this might explain the reduced VEGF level as we reported earlier [83]. 

## 5. The Epithelium as the Central Regulator of the Airway Wall

The epithelium separates the airway tissues from the inhaled air and, thus, protects the airways from damage and inflammation. The airway epithelium functions as a self-cleaning barrier through the secretion of mucus, which traps inhaled dust, chemicals, and microorganisms [84]. The mucus contains anti-microbial peptides, and the cilia move the mucus out of the airways into the esophagus from where it enters the digestive system to be degraded. 

In asthma, this protective function of the epithelium is disturbed, but it is unclear if this is the cause of the result of asthma. Airway epithelial cells are the first cell type to get into contact with pathogens and allergens. Airway epithelial cells not only separate the inhaled air from the lung tissues, but they also remove inhaled particles and produce a large number of chemokines and cytokines. Thus, epithelial cells communicate with immune cells and sub-epithelial tissue forming cells. Interestingly, several studies suggested that the airway epithelial cells have some sort of “memory” of previous infections or damages, which is linked to the immune response [43,44,84]. In another study, new variants of genes encoding for TNF receptors and TGF-β receptors were identified as risk factors for airway wall remodeling in asthma [85]. The composition of the extracellular matrix, especially of the basal membrane, was modified in childhood asthma, where epithelial cells produced insufficient amount of fibronectin and thereby reduced the capacity of the epithelium to repair [86]. 

The modified expression of these proteins affected the host response to infectious microorganisms, immune response, and tissue remodeling. The airway epithelium of the upper and lower respiratory tracts should be regarded as an integrated unit, which interacts on infections and chronic airway diseases through the epithelium. Specifically, the confirmation of the chromatin and protein folding controllers such as heat shock proteins (HSPs) were identified as essential contributors [87]. Moreover, HSP60 secreted by microorganisms such as *Chlamydia* have been shown 20 years ago to stimulate asthma exacerbation [88,89]. Airflow limitation correlated to *C. pneumoniae*-derived HSP60 in adult asthma patients [90]. Airway wall remodeling by *C. pneumoniae*-specific HSP60 involved toll like receptor 4 (TLR4) and p38 mitogen activated protein kinase (MAPK), followed by TGF-β activated kinase 1 [91]. As described above, the TGF-β signaling pathway is a well-known contributor to airway wall remodeling in asthma.

Circulating HSP60 and HSP70 have been suggested to play a role in asthma severity [92], which may be linked to an interaction between mother and child during embryogenesis [93]. A transcriptomic analysis further supported a role of HSP60 in macrophages of allergic asthma patients [94]. Airway fibroblast remodeling was sensitive to epithelial cell-derived HSP60, which increased the expression of an epigenetic regulator, protein arginine methyltransferase 1 (PRMT1) [95]. Earlier, it was shown that asthmatic airway smooth muscle cells constitutively expressed PRMT1 due to the lack of microRNA-19a [96]. In a further study, it was suggested that the ratio of epithelial cell-derived HSP60 to HSP70 and HSP90 affected airway wall remodeling by smooth muscle cells [97]. Moreover, the secretion of HSPs was sensitive to heat applied as a therapy for severe asthma during bronchial thermoplasty [95]. Remodeling parameters of human airway fibroblasts were also upregulated via PRMT1 and a signaling pathway involving C/EBP-β, leading to mitochondrial activity [98,99,100]. In airway epithelial cells, the processing of microRNAs might increase the susceptibility to develop asthma [101]. However, the role of the different HSPs and their variants in the context of asthma-associated airway wall remodeling is controversial and has to be further investigated [102]. 

The interaction between the epithelium and sub-epithelial mesenchymal fibroblasts and airway smooth muscle cells in the pathogenesis of asthma has been frequently discussed over decades, but rarely thoroughly studied [47]. The major problem for such research is the lack of valid in vitro models for human epithelial cells and mesenchymal cells. 

## 6. Parental Asthma, the, Environment, Epigenetics, and the Epithelium

**Figure 3:**
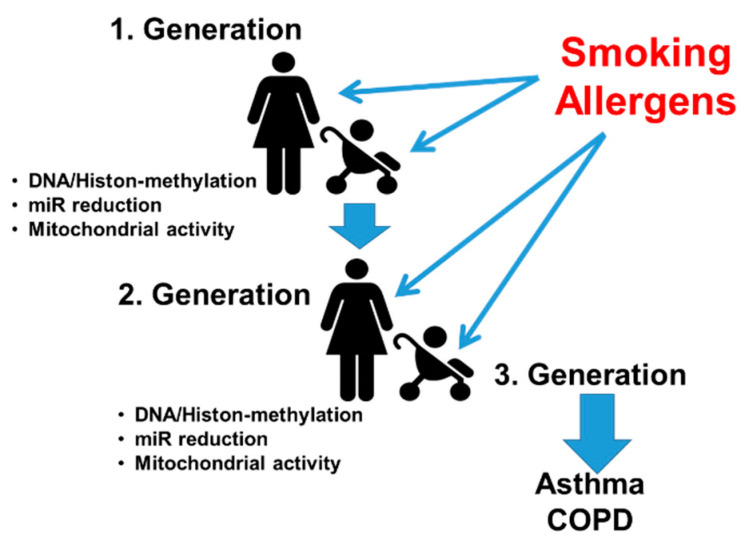
Epigenetic mechanisms may mimic genetic inheritance of asthma susceptibility. However, the reason as to how DNA and protein-methylation or the reduction of specific microRNAs (miR) become constitutive and inheritable remains to be investigated.

Epigenetic mechanisms such as methylation or acetylation have been linked to the pathogenesis of asthma as reviewed by Sheikhpour et al. [103], and can occur during embryogenesis by exposure of the mothers to environmental risk factors [104,105]. The exact mechanism(s) of how epigenetics makes the lungs more vulnerable to develop chronic inflammatory diseases later in life remains to be identified. Importantly, some of these epigenetic events can be handed down over several generations and seem to mimic genetic inheritance [106,107,108] (Figure 3).

Parental asthma, especially of mothers, is a well-recognized risk factor for childhood asthma, which cannot be fully explained by genetics or the shared environmental factors [109,110]. Specifically, greater influences are induced by uncontrolled maternal asthma during pregnancy [111]. Maternal atopy or asthma is associated with neonatal airway hyper-reactivity and impaired infant lung function, which is independent of allergen sensitization [112,113]. Exposure to low dose antibiotics during pregnancy has increased the risk of the child to develop asthma or eczema in the first 4 years after birth [114]. Animal studies demonstrated that offspring from transgenic mice with elevated eosinophil numbers and IL-5 levels generated had significantly higher nerve cell density in the airway epithelium, which might lead to airway hyper-responsiveness [21]. Hyper-innervation of the airway epithelium was due to impaired *in utero* lung development, which had been clearly linked to the onset of childhood asthma as reviewed by Belvisi [115]. Importantly, such structural alternations will remain unchanged until adulthood and, thus, represent a significant predisposition to asthma, as shown in a mouse model and humans [116].

In the DNA, clusters of cytosine and guanine dinucleotides, also named CpGs-island, were indicated as the preferred target for methylation [117]. A multi-cohort epigenome meta-analysis compared DNA-methylation in four age groups of asthmatics: (i) newborns, (ii) 4 years old, (iii) 16 years old, and (iv) adults [118]. This study identified 9 differentially methylated CpGs linked to asthma in newborns, and 36 CpGs linked to asthma in older children. Functional analysis of these epigenetic modifications confirmed the role of IL-5 (IL5RA) and potassium voltage gated channels (KCNH2) for the predisposition to asthma. However, the role of other novel biomarkers indicated in this study have to be confirmed in the future. Subsequent studies confirmed the important role of DNA methylation in the development of childhood asthma, as well as the potential to use the nasal epithelium as a source to identify early biomarkers for asthma [119]. Some of the genes harboring CpGs or other differentially methylated DNA sequences are related to epithelial cell function, epithelium integrity, and extracellular matrix remodeling, including: GJA4 (Gap junction protein alpha 4), POSTIN (Periostin), LDLRAD3 (low density lipoprotein receptor class A domain containing 3), ATP9B (Atpase Phospholipid transporting 9B), LAMA5 (Laminin subunit alpha 5), PDE6A (Phosphodiesterase 6A), NOS1AP(Nitric iodide synthase 1 adaptor protein), and KCNH2 (Potassium voltage-gated channel subfamily H member 2) [120,121,122,123]. All of these proteins have been linked to the presence of childhood asthma, but their detection requires tissue biopsies, which would be hard to obtain from children for diagnostic reasons.

## 7. The Difficulty to Study Airway Wall Remodeling, Particularly in Childhood Asthma

Two major obstacles make it difficult to study and understand airway wall remodeling in asthma: (i) the term “airway wall remodeling” is not well-defined and includes hyperplasia of the epithelium, increased basement membrane thickness, sub-epithelial extracellular matrix deposition, sub-epithelial fibrosis, and increased of smooth muscle mass; (ii) the classification of asthma pheno- and endo-types has been changed over the years. In addition, despite many studies, there is a lack of biomarkers that indicate airway wall remodeling, especially in childhood asthma [124]. Several studies aimed to link asthma pheno- and endo-types to different aspects of airway wall remodeling. Childhood asthma was subdivided into pheno- and endo-types according to: (i) epigenetic methylation of DNA and histones [125]; (ii) Th1 and Th2 cytokines [126,127]; (iii) comorbidities and response to treatment [128,129]; (iv) interferon γ/interleukin-5(IL-5)/IL-17 predominance [130]; (v) wheezing, atopy, and respiratory morbidity [131]. However, airway wall remodeling was not linked to any specific endo- or phenotype. It is therefore unclear if all aspects of airway wall remodeling are present in every patient, or if certain aspects are linked to different sub-types of asthma [6,29].

The hypothesis that the lung is pre-conditioned during embryogenesis and early childhood for the development of chronic inflammatory diseases later in life is gaining ground in the past years [132], especially with the observation that epigenetic events can become persistent [133]. Without a doubt, many, if not all, risk factors for asthma have been shown to initiate epigenetic modifications, but the mechanisms of how they become persistent remains unknown [134,135,136]. Thus, the major problems in studying and understanding airway wall remodeling in asthma are: (i) which asthma pheno-/endo-type to start with, (ii) which remodeling parameter(s) to focus on, and (iii) from whom to obtain patient samples.

As mentioned earlier, several environmental factors can initiate epigenetic modifications during embryogenesis or early in life. In regard to smoking-associated predisposition to asthma, the transmission of epigenetic mechanisms through mothers and grandmothers has been reported, as summarized in Figure 3 [137,138]. In addition, the use of e-cigarettes during pregnancy is also a cause of epigenetic modification of the embryo’s lung [139]. However, there is also indication that not only the mothers are responsible to “inherit” DNA-methylation patterns, but also fathers might be the origins of such modifications. DNA-methylation of the melatonin receptor has been described [140]. On the paternal side, in sperm, nicotine exposure has been reported to alter DNA-methylation of certain genes that are involved in lung development [141].

## 8. Conclusions

Airway wall remodeling is often a pathology already occurring in childhood asthma. The origin of airway wall remodeling in asthma is still unclear today. The above studies can be summarized as follows:

(1) It must be noted that a large range of environmental asthma risk factors such as cigarette smoke, fine dust, allergens, viruses, bacteria, etc., will initiate a protective response of the airway against these inhaled irritants. The available data suggest that this protective response of the airway wall is very similar, regardless of the nature of the trigger. However, future large cohort studies need to provide more evidence if specific types of tissue structural changes are unique for specific asthma endo- or phenotypes.
(2) There is evidence that pattern recognition receptors such as TLRs could explain how a wide range of different risk factors from the environment initiates airway wall remodeling during embryogenesis and early childhood. In adult asthma, some studies indicated that damage-associated molecular patterns and pathogens-associated molecular patterns play a role in tissue remodeling; however, this was so far only associated with age-related asthma [45]. It has not been investigated if these mechanisms might be active during embryogenesis and early childhood.
(3) Furthermore, it remains unknown why, for some people, this protective response does not shut down after the trigger is gone, and further leads to airway wall remodeling.
(4) Many cellular pathologies of airway wall remodeling in asthma are maintained in isolated cells; hence, indicating that the underlying mechanisms became persistent. Furthermore, these cell type specific pathologies of airway wall remodeling can be initiated by the above-named environmental asthma risk factors and the pattern recognition proteins through epigenetic events, including microRNA expression, DNA, and protein methylation/de-methylation.
(5) The epigenetic events can be passed over at least three generations, but the mechanism underlying this “inheritability” is unknown. Importantly, this “epigenetic inheritance” of the asthma predisposition might mimic real inheritance of susceptibility genes, which needs to be investigated.
(6) A major problem in detecting airway wall remodeling in childhood asthma is the lack of clear markers without obtaining tissue biopsies. This lack of information on the structural changes of the airways at early stages of asthma makes it difficult to correlate asthma pheno- and endo-types with specific aspects of remodeling.

There is increasing evidence that airway wall remodeling contributes to other asthma pathologies including inflammation, hyper-reactivity, and probably the development of allergies. Unfortunately, there is no form of therapy that can prevent, stop, or reverse airway wall remodeling. To find such therapies, it is essential to understand the pathogenesis of airway wall remodeling.
